# Supplementary material for: Assessment of the elite accessions of bael [Aegle marmelos (L.) Corr.] in Sri Lanka based on morphometric, organoleptic, and elemental properties of the fruits and phylogenetic relationships
Source: PLoS One. 2020 May 22;15(5):e0233609. doi: 10.1371/journal.pone.0233609 (PMC7244165; doi:10.1371/journal.pone.0233609)
Supplement: S1 Table — Initial Denaturation (I.D.); Denaturation (D); Primer Annealing (PA); Extension (E); Final Extension (F.E.). (DOCX) [file pone.0233609.s001.docx]

**S1 Table** DNA barcoding markers and PCR conditions

| **PCR Steps** | **State** | ***trnH-psbA*** | ***atpB-rbcL* spacer** | ***matK-trnT* spacer** | ***trn*L(*tRNA-leu*)** |
| --- | --- | --- | --- | --- | --- |
|  |  | P_F_: CGCGCATGGTGGATTCACAATCC  P_R_: GTTATGCATGAACGTAATGCTC | P_F_: GAAGTAGTAGGATTGATTCTC  P_R_: TACAGTTGTCCATGTACCAG | P_F_: GCATAAATATAYTCCYGAAARATAAGTGG  P_R_: TGGGTTGCTAACTCAATGG | P_F_: CGAAATCGGTAGACGCTACG  P_R_: GGGGATAGAGGGACTTGAAC |
| I.D. | Temp. | 98 ºC | 94 ºC | 95 ºC | 95 ºC |
|  | Time | 45 secs | 4 mins | 90 secs | 10 mins |
| D | Temp. | 98 ºC | 94 ºC | 95 ºC | 95 ºC |
|  | Time | 10 secs | 30 secs | 30 secs | 30 secs |
| PA | Temp. | 64 ºC | 45 ºC | 48 ºC | 50 ºC |
|  | Time | 30 secs | 30 secs | 1 min | 30 secs |
| E | Temp. | 72 ºC | 72 ºC | 68 ºC | 72 ºC |
|  | Time | 40 secs | 2 mins | 2 mins | 2 mins |
| F.E. | Temp. | 72 ºC | 72 ºC | 68 ºC | 94 ºC |
|  | Time | 10 mins | 5 mins | 20 mins | 4 mins |
| References | | [1, 2] | [3] | [4] | [5] |

Initial Denaturation (I.D.); Denaturation (D); Primer Annealing (PA); Extension (E); Final Extension (F.E.)

**References**

1. Tate JA, Simpson BB. Paraphyly of Tarasa (Malvaceae) and diverse origins of the polyploid species. Syst Bot. 2003; 28: 723-738. doi: 10.1043/02-64.1.

2. Sang T, Crawford DJ, Stuessy TF. Chloroplast DNA phylogeny, reticulate evolution, and biogeography of Paeonia (Paeoniaceae). Am J Bot. 1997; 84:1120-1136. doi: 10.2307/2446155.

3. Hoot SB, Taylor WC. The utility of nuclear *ITS*, a LEAFY homolog intron, and chloroplast *atpB-rbcL* spacer region data in phylogenetic analyses and species delimitation in Isoetes. Am Fern J. 2001; 91: 166-178. doi: 10.1640/0002-8444(2001)091[0166:TUONIA]2.0.CO;2.

4. Wicke S, Quandt D. Universal primers for the amplification of the plastid *trnK/matK* region in land plants. In: Anales del Jardín Botánico de Madrid. Consejo Superior de; 2009. pp. 285-288.

5. Taberlet P, Coissac E, Pompanon F, Gielly L, Miquel C, Valentini A, et al. Power and limitations of the chloroplast *trn L* (UAA) intron for plant DNA barcoding. Nucleic Acids Res, 2006; *35*e14-e14. doi: 10.1093/nar/gkl938.
