# Supplementary material for: Assessment of the elite accessions of bael [Aegle marmelos (L.) Corr.] in Sri Lanka based on morphometric, organoleptic, and elemental properties of the fruits and phylogenetic relationships
Source: PLoS One. 2020 May 22;15(5):e0233609. doi: 10.1371/journal.pone.0233609 (PMC7244165; doi:10.1371/journal.pone.0233609)
Supplement: S4 Table — (DOCX) [file pone.0233609.s004.docx]

**S4 Table** Details of the PCA for fruit size parameters

| **Criterion** | **PC1** | **PC2** | **PC3** |
| --- | --- | --- | --- |
| Eigen value | 4.64 | 0.25 | 0.11 |
| Proportion of variance explained | 0.93 | 0.05 | 0.02 |
| Cumulative Variance | 0.93 | 0.98 | 1.00 |
|  |  |  |  |
| **Variable** | **PC1** | **PC2** | **PC3** |
| Fruit weight (g) | -0.42 | 0.81 | 0.42 |
| Fruit length (cm) | -0.44 | 0.22 | -0.87 |
| Fruit width (cm) | -0.46 | -0.32 | 0.15 |
| Fruit circumference (cm) | -0.46 | -0.32 | 0.15 |
| Fruit inner diameter (cm) | -0.46 | -0.31 | 0.16 |
