# Supplementary material for: Assessment of the elite accessions of bael [Aegle marmelos (L.) Corr.] in Sri Lanka based on morphometric, organoleptic, and elemental properties of the fruits and phylogenetic relationships
Source: PLoS One. 2020 May 22;15(5):e0233609. doi: 10.1371/journal.pone.0233609 (PMC7244165; doi:10.1371/journal.pone.0233609)
Supplement: S5 Table — (DOCX) [file pone.0233609.s005.docx]

**S5 Table** Details of the PCA for fruit taste parameters

| **Criterion** | **PC1** | **PC2** | **PC3** |
| --- | --- | --- | --- |
| Eigen value | 3.98 | 1.47 | 0.53 |
| Proportion | 0.66 | 0.25 | 0.09 |
| Cumulative | 0.66 | 0.91 | 1.00 |
|  |  |  |  |
| **Variable** | **PC1** | **PC2** | **PC3** |
| External appearance | -0.38 | -0.47 | -0.42 |
| Flesh color | -0.23 | 0.73 | -0.07 |
| Aroma | -0.44 | 0.30 | -0.43 |
| Texture | -0.43 | -0.35 | 0.40 |
| Sweetness | -0.43 | 0.16 | 0.66 |
| Overall preference | -0.49 | -0.08 | -0.18 |
