# Supplementary material for: Assessment of the elite accessions of bael [Aegle marmelos (L.) Corr.] in Sri Lanka based on morphometric, organoleptic, and elemental properties of the fruits and phylogenetic relationships
Source: PLoS One. 2020 May 22;15(5):e0233609. doi: 10.1371/journal.pone.0233609 (PMC7244165; doi:10.1371/journal.pone.0233609)
Supplement: S6 Table — EV: Eigen value; PV: Proportion of variance; CV: Cumulative variance. (DOCX) [file pone.0233609.s006.docx]

**S6 Table** Details of the PCA for elemental contents

| **Criterion** | **PC1** | **PC2** | **PC3** | **PC4** | **PC5** | **PC6** | **PC7** | **PC8** | **PC9** | **PC10** | **PC11** | **PC12** | **PC13** | **PC14** |
| --- | --- | --- | --- | --- | --- | --- | --- | --- | --- | --- | --- | --- | --- | --- |
| EV | 9.939 | 6.440 | 5.966 | 4.297 | 2.205 | 1.747 | 1.400 | 0.764 | 0.706 | 0.539 | 0.473 | 0.364 | 0.097 | 0.064 |
| PV | 0.284 | 0.184 | 0.170 | 0.123 | 0.063 | 0.050 | 0.040 | 0.022 | 0.020 | 0.015 | 0.014 | 0.010 | 0.003 | 0.002 |
| CV | 0.284 | 0.468 | 0.638 | 0.761 | 0.824 | 0.874 | 0.914 | 0.936 | 0.956 | 0.971 | 0.985 | 0.995 | 0.998 | 1.000 |
|  |  |  |  |  |  |  |  |  |  |  |  |  |  |  |
| **Variable** | **PC1** | **PC2** | **PC3** | **PC4** | **PC5** | **PC6** | **PC7** | **PC8** | **PC9** | **PC10** | **PC11** | **PC12** | **PC13** | **PC14** |
| Li7 | -0.026 | -0.065 | 0.386 | -0.011 | 0.030 | 0.091 | -0.057 | -0.074 | -0.243 | -0.033 | -0.112 | 0.063 | 0.095 | -0.179 |
| Be9 | 0.011 | -0.030 | 0.366 | -0.103 | -0.006 | -0.002 | 0.232 | -0.253 | 0.068 | -0.041 | 0.092 | -0.179 | -0.211 | 0.154 |
| Na23 | 0.261 | -0.104 | 0.112 | -0.039 | 0.164 | -0.093 | -0.159 | 0.117 | -0.107 | 0.042 | -0.031 | 0.264 | -0.392 | 0.117 |
| Mg24 | -0.116 | 0.190 | 0.146 | 0.328 | -0.104 | 0.047 | 0.009 | -0.118 | 0.024 | -0.048 | -0.032 | 0.056 | -0.146 | 0.064 |
| Al27 | -0.220 | 0.201 | -0.087 | 0.001 | -0.195 | 0.034 | -0.172 | 0.005 | 0.304 | 0.015 | -0.097 | 0.173 | -0.250 | 0.113 |
| K39 | 0.209 | 0.246 | 0.009 | -0.181 | 0.005 | -0.107 | 0.003 | -0.094 | 0.045 | 0.074 | -0.001 | 0.031 | 0.025 | -0.193 |
| Ca44 | 0.194 | 0.279 | 0.004 | -0.150 | -0.008 | -0.083 | -0.021 | -0.062 | 0.091 | 0.061 | -0.023 | 0.076 | 0.006 | -0.167 |
| Ti48 | -0.123 | 0.305 | 0.126 | 0.121 | 0.173 | 0.090 | -0.018 | -0.057 | 0.037 | -0.014 | 0.124 | 0.020 | 0.047 | -0.152 |
| V51 | -0.186 | 0.031 | 0.243 | -0.129 | 0.227 | 0.050 | 0.086 | 0.115 | 0.008 | -0.294 | 0.270 | 0.104 | -0.072 | -0.187 |
| Cr52 | 0.033 | 0.052 | -0.267 | -0.020 | 0.306 | -0.349 | 0.173 | 0.040 | -0.193 | 0.308 | -0.056 | 0.036 | -0.180 | -0.105 |
| Mn55 | -0.248 | 0.057 | -0.134 | -0.113 | 0.229 | -0.114 | 0.134 | 0.207 | -0.086 | -0.033 | -0.019 | 0.046 | -0.017 | 0.155 |
| Fe56 | -0.247 | 0.064 | -0.137 | -0.117 | 0.226 | -0.115 | 0.136 | 0.197 | -0.077 | -0.027 | -0.032 | 0.039 | -0.036 | 0.157 |
| Fe57 | -0.281 | -0.075 | -0.056 | -0.131 | 0.080 | -0.056 | 0.064 | -0.149 | -0.166 | 0.120 | -0.153 | -0.006 | 0.010 | 0.249 |
| Co59 | -0.286 | -0.029 | 0.036 | -0.045 | -0.035 | 0.080 | 0.072 | -0.318 | 0.251 | 0.166 | -0.105 | 0.044 | 0.046 | -0.047 |
| Ni60 | -0.277 | -0.096 | -0.093 | -0.064 | 0.067 | -0.008 | 0.046 | -0.285 | -0.001 | -0.014 | -0.119 | -0.055 | -0.410 | -0.346 |
| Cu63 | -0.064 | 0.322 | 0.067 | -0.241 | 0.030 | 0.031 | 0.002 | 0.011 | -0.063 | 0.076 | 0.001 | 0.099 | 0.086 | -0.095 |
| Zn66 | 0.099 | 0.059 | 0.285 | 0.162 | 0.118 | -0.130 | 0.326 | -0.125 | 0.017 | 0.131 | -0.314 | 0.042 | -0.019 | -0.288 |
| Ga71 | 0.012 | 0.250 | -0.007 | -0.217 | -0.193 | 0.031 | -0.161 | -0.336 | -0.362 | 0.380 | -0.004 | -0.186 | -0.040 | 0.238 |
| As75 | 0.185 | -0.059 | 0.205 | 0.056 | 0.265 | -0.075 | 0.250 | -0.184 | 0.230 | -0.023 | 0.200 | -0.275 | -0.020 | 0.398 |
| Se77 | -0.177 | -0.076 | 0.231 | -0.086 | 0.027 | 0.107 | 0.142 | 0.186 | 0.310 | 0.391 | -0.017 | 0.440 | -0.050 | 0.189 |
| Se78 | -0.072 | 0.291 | 0.139 | -0.232 | -0.030 | -0.069 | -0.002 | 0.196 | -0.043 | -0.121 | 0.066 | 0.034 | 0.192 | -0.050 |
| Se82 | 0.151 | 0.258 | -0.158 | -0.004 | -0.189 | -0.120 | 0.127 | -0.029 | 0.284 | -0.060 | 0.105 | 0.093 | -0.047 | 0.013 |
| Rb85 | 0.267 | 0.128 | 0.091 | -0.019 | -0.003 | 0.006 | -0.268 | 0.008 | 0.095 | 0.036 | -0.010 | 0.226 | -0.265 | 0.165 |
| Sr88 | 0.092 | 0.117 | -0.039 | -0.186 | -0.155 | 0.348 | 0.333 | -0.015 | -0.260 | -0.452 | -0.401 | 0.169 | -0.177 | 0.209 |
| Ag107 | -0.004 | -0.064 | 0.153 | 0.127 | -0.418 | -0.425 | 0.133 | 0.138 | -0.135 | 0.003 | -0.150 | -0.002 | -0.078 | -0.033 |
| Cd111 | -0.130 | -0.014 | 0.249 | 0.187 | -0.274 | -0.165 | 0.105 | 0.258 | -0.090 | 0.165 | -0.106 | 0.033 | 0.208 | 0.092 |
| In115 | 0.174 | 0.127 | -0.182 | -0.026 | -0.101 | -0.234 | 0.430 | -0.010 | 0.110 | -0.102 | 0.087 | -0.018 | -0.017 | 0.026 |
| Cs133 | 0.234 | -0.166 | 0.162 | -0.010 | 0.166 | -0.056 | -0.114 | 0.057 | -0.181 | 0.027 | 0.008 | 0.127 | -0.119 | -0.012 |
| Ba137 | 0.079 | -0.011 | -0.082 | 0.048 | -0.184 | 0.506 | 0.311 | 0.349 | -0.101 | 0.360 | 0.257 | -0.227 | -0.300 | -0.177 |
| Hg202 | 0.252 | 0.032 | -0.019 | -0.092 | 0.201 | 0.272 | 0.161 | -0.033 | 0.024 | 0.214 | -0.220 | 0.123 | 0.392 | 0.026 |
| Tl205 | -0.015 | 0.223 | -0.046 | 0.374 | 0.144 | 0.075 | -0.015 | -0.006 | -0.079 | 0.008 | -0.073 | 0.024 | -0.057 | -0.030 |
| Pb206 | -0.015 | 0.212 | -0.046 | 0.383 | 0.152 | 0.073 | -0.008 | 0.008 | -0.063 | -0.008 | -0.073 | 0.032 | -0.055 | -0.040 |
| Pb207 | -0.021 | 0.215 | -0.067 | 0.374 | 0.150 | 0.060 | -0.014 | -0.008 | -0.098 | -0.015 | -0.042 | -0.026 | 0.069 | 0.279 |
| Pb208 | -0.009 | 0.188 | 0.189 | -0.153 | 0.138 | -0.017 | -0.198 | 0.370 | 0.243 | 0.011 | -0.427 | -0.585 | -0.146 | 0.023 |
| Bi209 | -0.127 | 0.251 | 0.219 | -0.031 | -0.034 | -0.082 | 0.009 | 0.013 | -0.274 | 0.014 | 0.400 | -0.007 | -0.068 | 0.084 |
| U238 | -0.026 | -0.065 | 0.386 | -0.011 | 0.030 | 0.091 | -0.057 | -0.074 | -0.243 | -0.033 | -0.112 | 0.063 | 0.095 | -0.179 |

EV: Eigen value; PV: Proportion of variance; CV: Cumulative variance
