# Supplementary figures and images for: Assessment of the elite accessions of bael [Aegle marmelos (L.) Corr.] in Sri Lanka based on morphometric, organoleptic, and elemental properties of the fruits and phylogenetic relationships
Source: PLoS One. 2020 May 22;15(5):e0233609. doi: 10.1371/journal.pone.0233609 (PMC7244165; doi:10.1371/journal.pone.0233609)

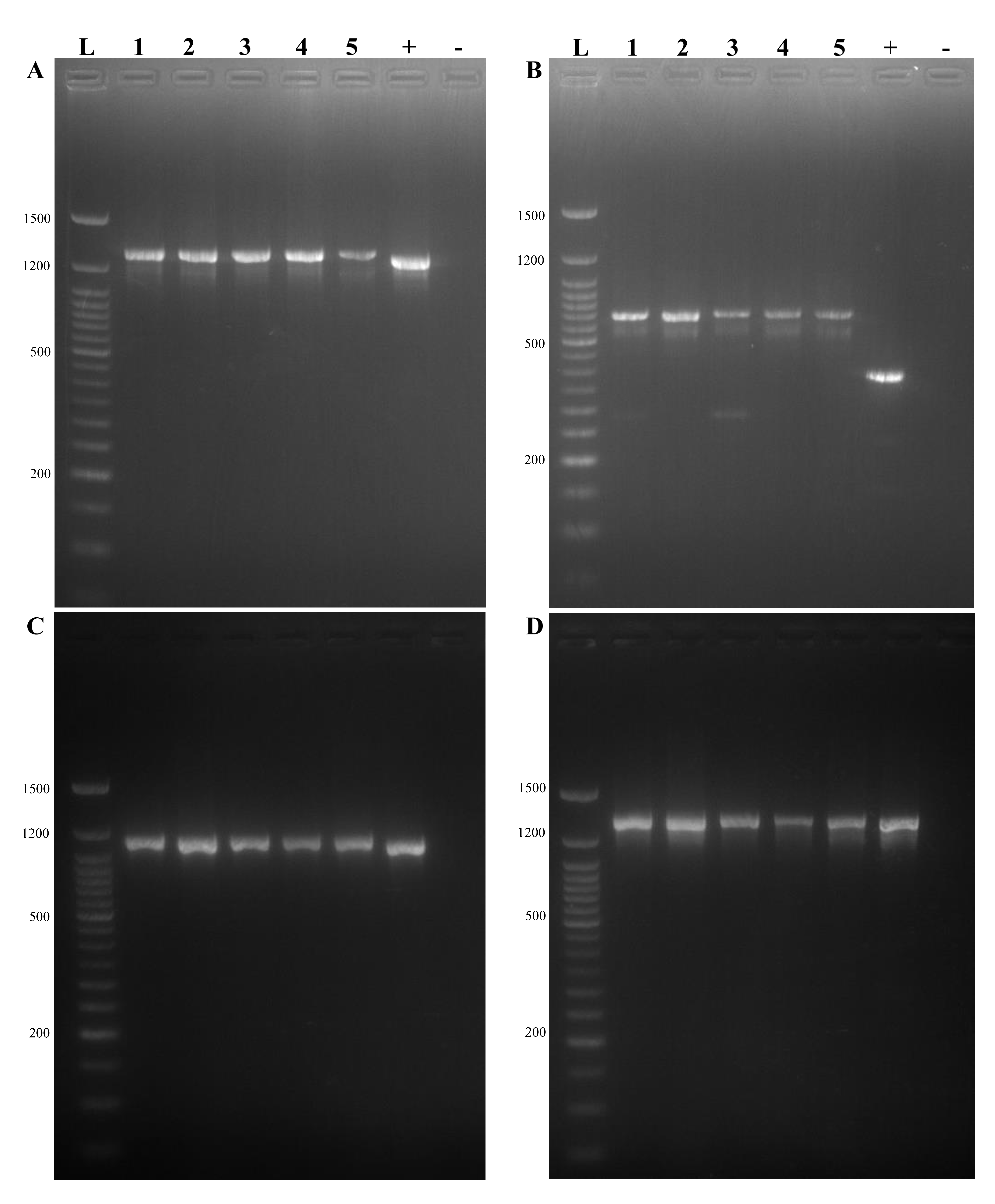

Supplement: S1 Fig — A: atpB-rbcL; B: trnH-psbA; C: matk-trnT; D: tRNA-leu. L: 50 bp ladder, 1: Beheth Beli (BB); 2: Paragammana (PA); 3: Mawanalla (MA); 4: Rambukkana (RA); 5: Polonnaruwa-Supun (PS); +: positive control (DNA of the apple variety Spartan); -: negative control (PCR mixture without template DNA). (TIF) [file pone.0233609.s007.tif]

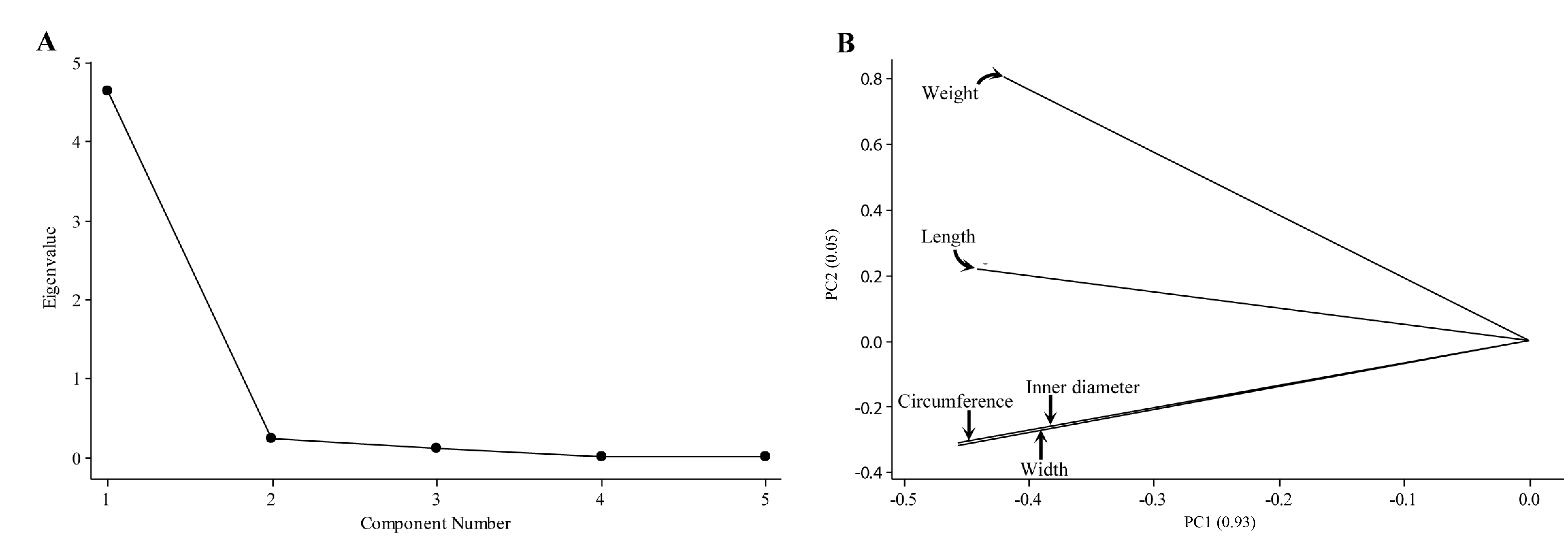

Supplement: S2 Fig — A: Scree plot; B: Loading plot. The contribution of the PC to the total variance is given in parenthesis. (TIF) [file pone.0233609.s008.tif]

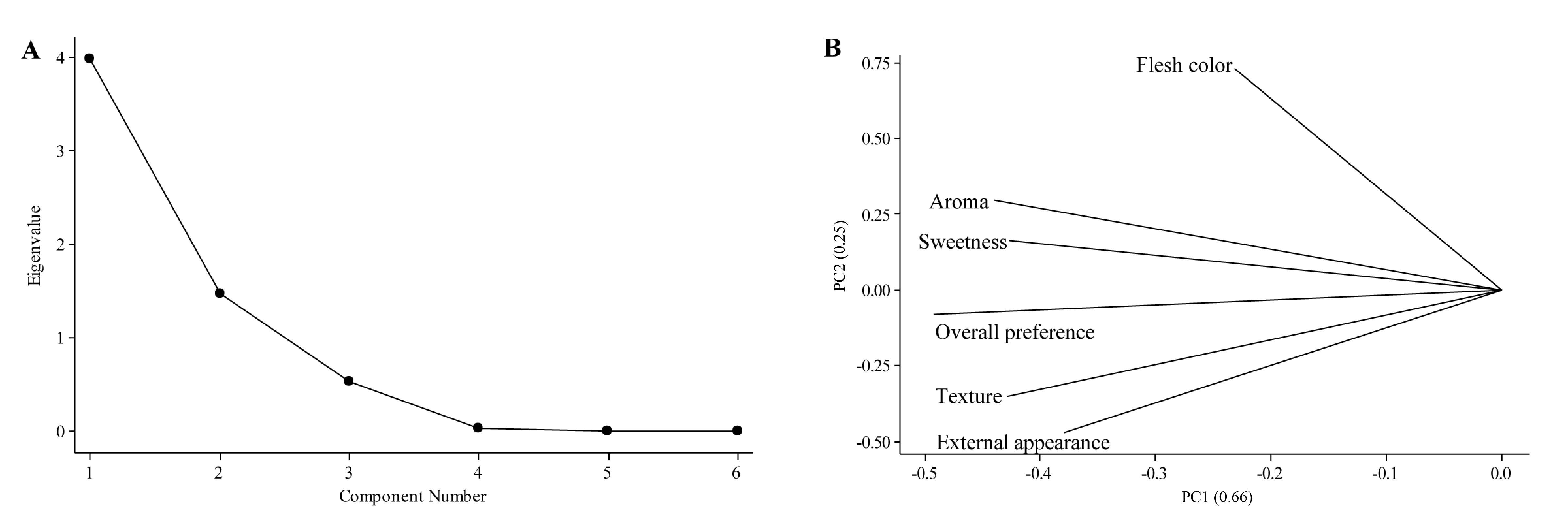

Supplement: S3 Fig — A: Scree plot; B: Loading plot. The contribution of the PC to the total variance is given in parenthesis. (TIF) [file pone.0233609.s009.tif]

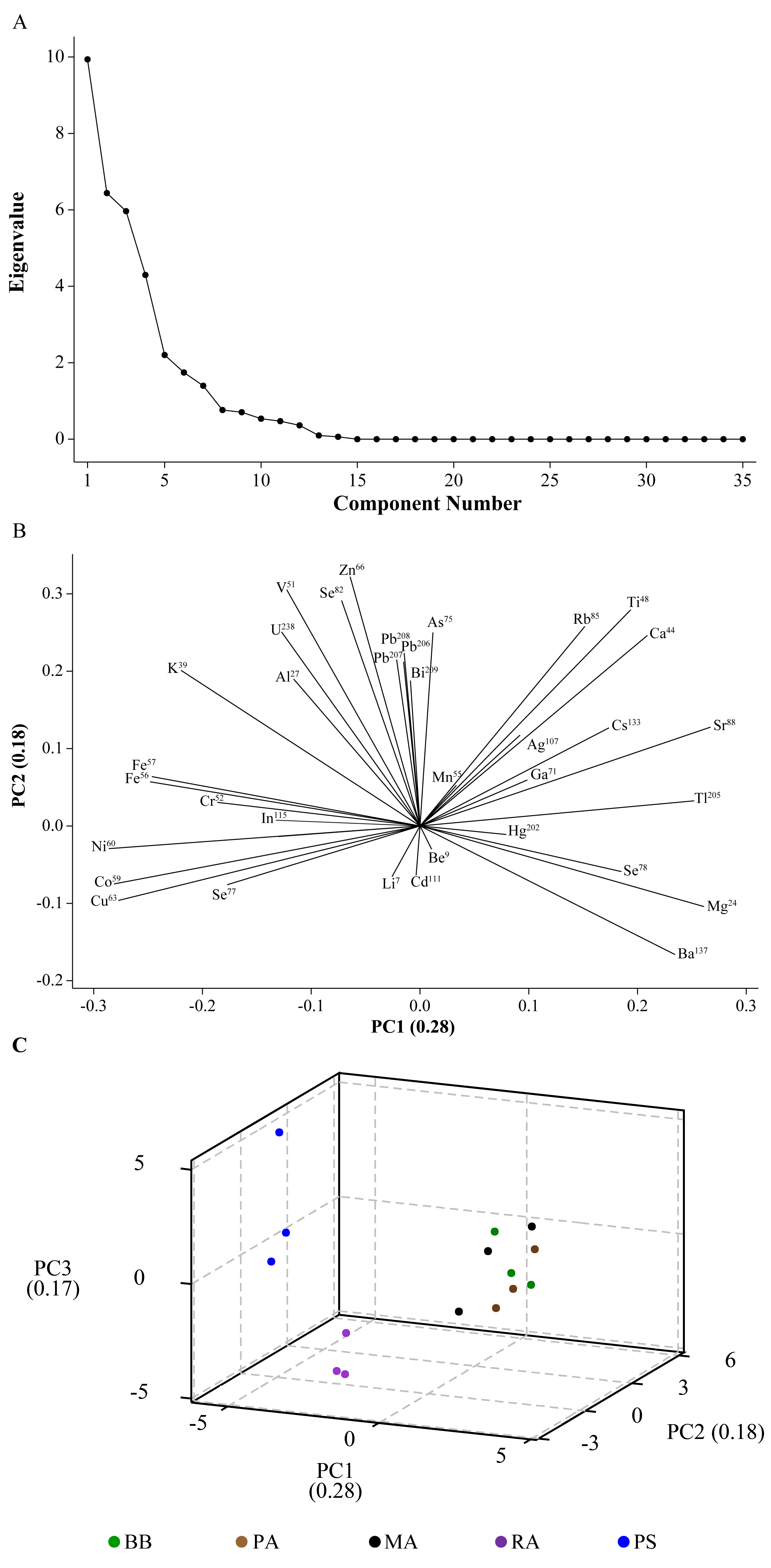

Supplement: S4 Fig — A: Scree plot; B: Loading plot. The contribution of the PC to the total variance is given in parenthesis. (TIF) [file pone.0233609.s010.tif]
